# Use of Live Biopreservatives and Bacteriophages to Enhance the Safety of Meat Products

**DOI:** 10.3390/life15020197

**Published:** 2025-01-28

**Authors:** Cristina Rodríguez-Marca, Cristina Domenech-Coca, Miho Nakamura, Nàdia Ortega-Olivé, Pere Puigbò

**Affiliations:** 1 Nutrition and Health Unit, Eurecat, Technology Centre of Catalonia, 43204 Reus, Catalonia, Spain; cristina.rodriguez@eurecat.org; 2 Department of Biochemistry and Biotechnology, University Rovira i Virgili, 43007 Tarragona, Catalonia, Spain; cristina.domenech@urv.cat; 3 Medicity Research Laboratory, Faculty of Medicine, University of Turku, 20520 Turku, Finland; miho.nakamura@utu.fi; 4 Institute of Biomaterials and Bioengineering, Tokyo Medical and Dental University, Tokyo 101-0062, Japan; 5 Graduate School of Engineering, Tohoku University, Sendai 980-8577, Japan; 6 Department of Food Technology, Engineering and Science, University of Lleida, 25198 Leida, Catalonia, Spain; nadia.ortega@udl.cat; 7 Department of Biology, University of Turku, 20014 Turku, Finland; 8 Department of Animal and Food Science, Autonomous University of Barcelona, 08193 Bellaterra, Catalonia, Spain

**Keywords:** live biopreservatives, bacteriophages, lactic acid bacteria, meat safety, nitrites, bacteriocins, natural antimicrobials, biodegradable food packaging, foodborne pathogens, environmental microbes, microevolutionary models, interaction models

## Abstract

Critical health considerations for both raw and processed meats include addressing bacterial spoilage and ensuring safety. Nitrites and nitrates are widely used in the meat industry to enhance color and flavor and extend shelf life. However, health concerns linked to their use make reducing nitrites and nitrates in meat production a significant challenge with potential benefits for both the food industry and consumer health. This challenge has been addressed with the use of biopreservatives, i.e., substances extracted from natural sources or produced by fermentation that can enhance food quality and safety. In this article, we assess the use of live biopreservatives (LBs), defined here as microorganisms that produce antimicrobial substances that can be used to preserve and extend the shelf life of food. Moreover, the potential synergistic effects of LBs with bacteriophages and biodegradable food packaging for meat is also explored. This innovative combination offers a comprehensive approach to meat preservation, enhancing both microbial control and sustainability. Overall, the inclusion of LBs extends the shelf life of meat products through bacteriostatic mechanisms, whereas bacteriophages offer direct (lytic) action against pathogens. Enhancing meat preservation and safety with mixed microbe-mediated strategies requires deeper empirical and theoretical insights and further revision of laws and ethical considerations.

## 1. Introduction

The food industry faces a significant challenge in controlling foodborne pathogens in meat and meat products [1]. Bacterial spoilage and safety are critical health concerns for both raw and processed meats. [2,3]. The use of nitrites and nitrates as preservatives in the meat industry has been associated with various functions, such as enhancing color and flavor and extending shelf life [1]. However, there are concerns regarding their safety due to the formation of potentially carcinogenic compounds called N-nitrosamines [1]. The European Food Safety Authority (EFSA)’s 2023 report warned about the potential DNA-damaging and carcinogenic effects of 10 nitrosamine substances that are predominantly present in processed and cured meat products [4]. However, technological changes are necessary to eliminate nitrates/nitrites and ensure the production of high-quality salamis while lowering the ripening temperature to ensure microbiological safety [5]. Thus, natural sources and new technologies are being explored to develop alternative additives [6]. Reducing the use of nitrites and nitrates in meat production is a major challenge that should substantially benefit the food industry and consumer health.

It has been suggested that these challenges can be successfully addressed by strategically utilizing biopreservatives [1], i.e., natural substances derived from bacteria, fungi, plants or animals that can be used to extend the shelf life of food products while guaranteeing their safety [7]. Natural preservatives offer a promising alternative to combat microbial resistance [8] and mitigate the adverse effects associated with certain synthetic compounds [7]. Moreover, studies on the mechanism of action, safety evaluations, and the establishment of appropriate regulations for natural preservatives are needed before they are used [1].

## 2. Antimicrobial Innovations

Antimicrobial innovations encompass the development and implementation of novel strategies, compounds, and technologies designed to prevent and control the growth of harmful microorganisms [9]. With the rise of antimicrobial resistance, these innovations are essential to protect people’s health, extend products’ shelf lives, and reduce reliance on traditional antibiotics [10]. Research on antimicrobial innovations calls for actions that offer a comprehensive assessment of novel biopreservation strategies, such as plant-based extracts, peptides, nanomaterials, and bacteriophages [11]. Each of these approaches offers unique advantages in addressing microbial threats and combating the spread of antimicrobial resistance. This review aims to provide guidance regarding all aspects that should be thoroughly addressed in the special issue. In this article, we provide an overview of the critical intersection between live biopreservatives (LBs), bacteriophages, and biodegradable food packaging for meat biopreservation, addressing key considerations from scientific, technological, and socioeconomic perspectives. Furthermore, we provide a theoretical and a mathematical model that can be used as guidance to quantify the effect of LBs and bacteriophages in real-world scenarios.

## 3. Use of Biopreservatives to Enhance Food Quality and Safety

Biopreservatives are antimicrobial substances extracted from natural sources or produced by fermentation that enhance food quality and safety [12]. Bacteriocins are antimicrobial peptides produced by microorganisms like LAB to gain a competitive advantage in their environment, while being harmless to the producing strains [13]. These biologically active compounds include peptide structures that exhibit antimicrobial activity [14]. Bacteriocins are naturally produced by certain bacterial strains; however, their use as additives may be limited by their high cost and limited effectiveness against specific pathogenic microorganisms in the final product [1]. Among all the purified bacteriocins tested thus far, nisin (E-234) stands out as the only one permitted as a food additive in the European Union, as stated by the European Commission in 2011 [1,7]. Furthermore, Pediocin PA-1, a crude extract obtained from bacteriocin-producing strains, is also available commercially. Although nisin has relatively broad-spectrum antimicrobial properties, including the inhibition of the overgrowth of sporulated bacteria, these agents are not effective in meat production [2]. Bacteriocins and bacteriocin-producing bacteria have been proposed for use as potential natural preservatives [15]. However, the major effect of bacteriocins on food is obtained when they are combined with other preservation techniques [16] such as the use of essential oils (EOs). A wide range of EO applications, such as incorporation in active packaging or edible films and direct encapsulation, have been described [16]. The combined use of EOs and bacteriocins or bacteriocin-producing bacteria can not only improve food safety and the shelf lives of meat and meat products, but also stabilize sensory and nutritional quality [16,17].

## 4. Live Biopreservatives (LBs)

**Figure 1:**
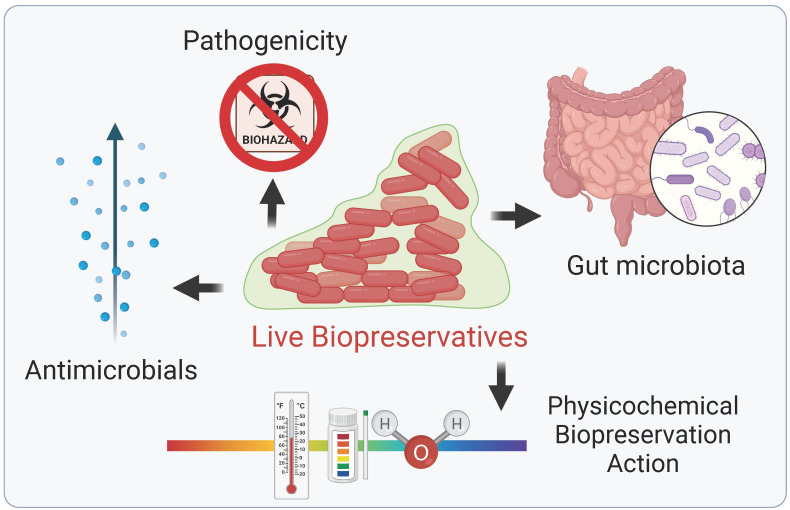
Main characteristics of live biopreservatives (LBs). LBs are microbial strains that can be utilized as food preservatives because they have the capacity to secrete antimicrobial substances (such as lactic and acetic acids and bacteriocins). Furthermore, they need to present the following desirable characteristics: antimicrobial properties; absence of pathogenicity elements; limited impact on product characteristics; biopreservation action spanning a wide range of temperatures, water activity and pH levels; and lower impact on the gut microbiota.

LBs are defined here as microorganisms capable of secreting antimicrobial substances that can be utilized as a way of preserving food and extending its shelf life (Figure 1). LBs have a bacteriostatic effect; i.e., they prevent the growth of bacteria. LBs possess diverse characteristics beyond their antimicrobial properties [18]. These include minimal potential for pathogenic activity, in compliance with regulatory protocols; limited impact on product characteristics, such as sensory attributes and color [18]; biopreservation action spanning a wide range of temperatures and pH levels [14,19]; and versatile activity against a spectrum of microorganisms [20]. LBs also exhibit resistance through mechanisms of action that impede microbial adaptation [21]. Furthermore, LBs may have a lower impact on the gut microbiota than synthetic preservatives [22]. The potential for a decreased impact on beneficial bacteria in the gut makes LBs an attractive option for those concerned about the effects of food preservatives on overall gut health.

## 5. Lactic Acid Bacteria to Enhance Meat Product Safety

Research on meat protection has focused on the selection of lactic acid bacteria (LAB) that do not cause meat spoilage prevents meat spoilage and that enhance product safety [2]. However, the use of LAB is controversial because they can either contribute to spoilage (through the generation of harmful metabolites and the subsequent organoleptic downgrading of meat) or act as stabilizing agents that prevent the growth of other harmful bacteria [23]. In addition to LAB, *Pseudomonas* species have been suggested to be key organisms for consideration when evaluating the impact of specific chilling durations and temperature conditions on the growth of spoilage bacteria [24]. Certain probiotics have also been utilized as LBs in the preservation of fermented meat products [25,26,27].

LAB protect meats by outcompeting other microorganisms and producing inhibitory substances such as lactic and acetic acids and bacteriocins [2,13,28,29]. LAB-preservation methods, such as spray drying, are effective methods that allow products to be obtained at a low cost compared with lyophilization [30]. Ongoing scientific efforts have led to the emergence of a growing market for food products that incorporate protective cultures to enhance food safety and extend shelf life [31]. Notably, the Lactobacillales order, which includes various genera, plays a significant role in this context; for example, a starter culture (*Latilactobacillus sakei* CTC494) has been investigated for its ability to protect against *Listeria monocytogenes* in two varieties of chicken-based dry-fermented sausages during fermentation and ripening [32]. Moreover, mathematical models are also utilized in anti-Listeria biopreservation strategies [33].

Several studies have tested the effectiveness of combining different LAB or their metabolites with novel preservative techniques, such as gas packing, natural extracts, essential oils, and bacteriocins, to create more selective and defensive systems to overcome pathogenic and spoilage microorganisms [34] while also enhancing the physicochemical and sensory properties of the bacteria. Specifically, mixed methods, such as the adoption of higher concentrations of bioprotective starter cultures along with a low sugar concentration, have been shown to improve textural features and reduce oxidation levels in salamis subjected to challenges with *Listeria innocua*, *Salmonella enterica*, and *Clostridium botulinum* [5]. Furthermore, LAB can promote the decomposition of proteins and lipids to produce flavor-precursor substances such as free amino acids or free fatty acids, give food a unique flavor, and have a certain positive impact on the overall flavor of the finished product (they thus yield flavor improvement along with product preservation and, ultimately, the extension of shelf life [35]). LAB play a key role in the production of fermented meat products, resulting in texture and flavor improvement, product preservation and, ultimately, the extension of shelf life [34].

## 6. Bacteriophages to Enhance Food Safety

Bacteriophages, also known as phages, are the most abundant organisms in the biosphere and replicate in bacterial cells [36]. Bacteriophages are naturally occurring predators of bacteria and are presumably harmless to humans and animals; thus, they have been utilized as antimicrobial agents in food products [37,38,39,40,41]. Lytic bacteriophages, with their strict host specificity, are promising biocontrol agents for specifically targeting and controlling pathogenic bacteria in the food industry [42]. Moreover, natural and genetically modified bacteriophages may also play a future role in meat preservation [15], preventing pathogen growth and increasing shelf life. The role of bacteriophages includes the control of the following bacterial pathogens: (i) *L. monocytogenes*: bacteriophages have recently been used commercially for pathogen biocontrol in the food industry (in meat and poultry products) [37]; (ii) *Salmonella* sp.: bacteriophages have also been used in ground beef [43], ground pork [43] and fresh-cut fruits under different storage temperatures; and (iii) Shiga toxin-producing *Escherichia coli* (STEC) [44]. Furthermore, bacteriophages have been employed as effective biomarkers for fecal contamination at various processing stages in poultry and beef [38].

While recent advancements in biological interventions suggest bacteriophages have potential for use as effective microbial regulators, further research is needed to optimize their use as antimicrobial agents [38]. This includes determining the threshold level of hosts necessary for efficient bacteriophage replication and identifying the optimum temperature conditions that enhance bacteriophage performance [38]. A common practice in phage therapy, i.e., the use of phages for the treatment of pathogenic bacterial infection, is to use phage therapy in combination with antibiotics that can interact synergistically [45]. A parallel approach would involve incorporating bacteriophages into mixed strategies for food preservation, combining the use of LBs to prolong shelf life through bacteriostatic mechanisms with the use of bacteriophages to provide direct (lytic) action against pathogens. However, a more comprehensive empirical and theoretical understanding of microbe-mediated strategies, specifically of those incorporating LBs and bacteriophages, is necessary to effectively address challenges in meat preservation.

## 7. Biodegradable Food Packaging for Meat Products

Spoilage control in meat and meat products presents a critical challenge in the food industry due to the high water content, water activity, pH, and nutrient availability in meat [34]. For this reason, specific packaging materials are needed to ensure food safety and product stability in terms of water content and oxidation. Maintaining color stability in fresh meat requires gas-permeable films, whereas gas-barrier films are preferred to prevent the oxidation of fats and proteins in meat products [46]. However, petroleum-derived materials used for meat packaging are becoming obsolete because they are not environmentally sustainable since the European Union (EU) adopted the strategy of making all plastic packing recyclable or reusable in the EU market by 2030. This strategy aims to protect our environment and reduce marine litter and greenhouse gas emissions, and dependence on fossil fuels is considered a key element of Europe’s transition to a carbon-neutral and circular economy [47].

Furthermore, environmental awareness has increased in recent years. Consumers demand new ecologically friendly and sustainable ways of preserving food [1]. In this context, biodegradable materials obtained from natural sources represent an alternative in meat production and packaging, although they do not have sufficient mechanical and barrier properties. These biopolymers can be categorized based upon the source of the material. The most well-known natural biopolymers are polysaccharides and proteins such as starch, cellulose, chitosan, and gelatin [46]. On the other hand, some synthetic biopolymers, e.g., polyhydroxyalkanoate (PHA) and polyhydroxybutyrate (PHB), can be generated by fermentation, while others, such as polylactic acid (PLA), can be produced by chemical synthesis from biomass [46].

Current research on natural biopolymers is focused on developing biodegradable materials from agrifood byproducts as a strategy to reduce environmental impact and contribute to a circular economy. This provides an opportunity for agricultural and food industries to revalorize byproducts and create added value in the market. Although biodegradable food packaging for meat [48,49,50] has been used for a long time, the use of microbial biopreservatives [23] represents an unexplored challenge in the field. In this context, LAB are highly interesting due to their ability to be encapsulated by extrusion to produce antimicrobial films [20]. Therefore, as soon as biodegradable materials overcome current technical limitations, additional studies will be needed to validate the integration of microbial bioprotectors in emergent materials. Furthermore, to the best of our knowledge there are no studies that integrate the use of LBs and bacteriophages with that of upcycled agrifood for packaging.

## 8. Future Perspectives

To address future challenges in the use of LBs and bacteriophages, both short-term and long-term goals are essential. In the short term, the application of LAB and bacteriocins is ready for real-world scaling (Section 8.2). In the mid-term, computational models are required to evaluate the implementation of bacteriophages in the meat industry (Section 8.2) and assess the potential impact of LBs and bacteriophages on the global antimicrobial-resistance crisis (Section 8.3). In the long term, the integration of LBs and bacteriophages into various types of biodegradable packaging (Section 8.4) and the evaluation of their economic and environmental impacts are key objectives.

### 8.1. Scaling Up: Use of LAB and Bacteriocins in Real Meat Products

**Figure 2:**
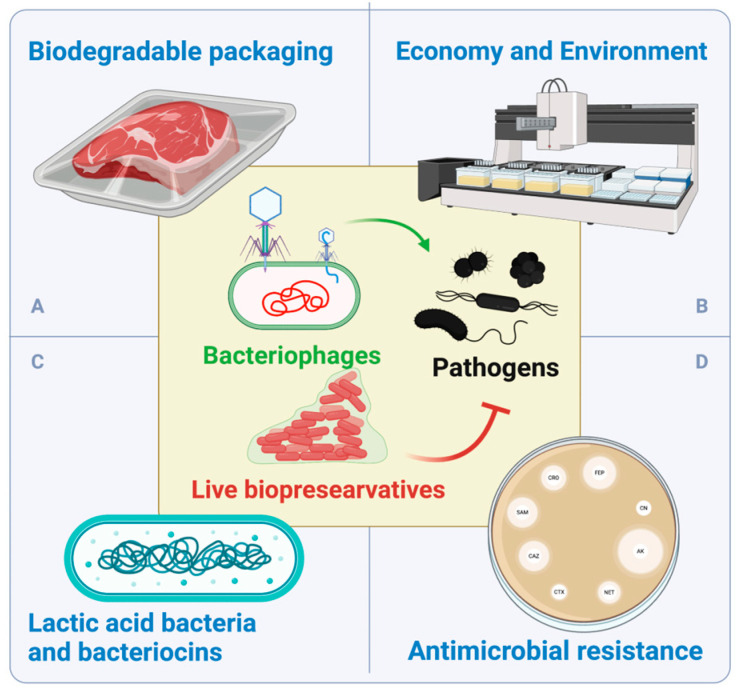
Future perspectives. The utilization of live biopreservatives (LBs) as bacteriostatic agents, combined with the use of lytic bacteriophages, poses future research and innovation challenges for the meat industry. (**A**) Biodegradable food packaging has been in the market for a few years already. However, its potential interactions with LBs are not yet fully understood. Moreover, the combination of LBs (bacteriostatic effect), bacteriophages (bacteriolytic effect), and biodegradable food packaging has never been tested. (**B**) A reduction in the use of nitrites and nitrates will bring potential benefits to the food industry, the environment, and consumer health. However, before they can reach the market, LBs require further investigations into their economic challenges. Furthermore, bacteriophages are not yet regulated as biopreservatives by the major regulatory agencies, including those in the European Union and the United States. (**C**) Lactic acid bacteria and bacteriocins have performed well in controlled settings. However, antimicrobial agents need to be scaled up to demonstrate their usability in highly complex meat matrices. (**D**) Antimicrobial resistance is a major global challenge. Interspecific horizontal gene transfer (HGT), including the exchange of antimicrobial resistance genes, is pervasive in bacteria. Future experiments on the use of LBs and bacteriophages need to explore the potential highways for HGT that can be created between LBs and environmental microbes.

The future integration of LAB and bacteriocins in real meat products holds substantial promise for revolutionizing the food industry (Figure 2C). Despite their proven efficacy in in vitro settings, the full potential of these natural antimicrobial agents in real meat applications remains largely untapped due to complexities inherent in meat matrices [1]. The approval of nisin as a purified bacteriocin additive in European and US food regulations [1] and the commercial availability of crude extracts, such as Pediocin PA-1 from bacteriocin-producing strains, highlights its potential as an effective biopreservative [51,52].

Advancements in understanding the behavior of LAB and bacteriocins in different meat environments are shaping tailored approaches. For instance, *Pseudomonas* species thrive in aerobic environments, while LAB flourish in anaerobic settings, such as in vacuum-packed meat [24]. This knowledge aids in optimizing the effectiveness of these products against specific spoilage or pathogenic microorganisms present in different meat types. Moreover, the use of LAB cocktails or synergistic combinations of bacteriocins has the potential to create robust antimicrobial systems that can combat a broader spectrum of microbes, ensuring product safety and quality of meat products, as well as prolonging shelf life and preserving sensory attributes [7,53]. Innovations in processing technologies, such as controlled fermentation temperatures, targeted bioprotective starter cultures, and emerging techniques such as high-pressure processing (HPP), are also proving instrumental in harnessing the antimicrobial capabilities of LAB and bacteriocins [20].

Looking ahead, the future landscape of meat preservation and safety is likely to shift toward greater reliance on natural antimicrobial agents such as LAB and bacteriocins. As research and technological advancements continue to unfold, the effective integration of these natural compounds in meat processing is advised to address food-safety concerns, extend shelf life, and satisfy consumer demands for cleaner labels and natural preservatives in meat products. This evolution represents a paradigm shift toward sustainable and innovative food-preservation methodologies in the meat industry.

### 8.2. Computational Models for Assessing the Use of LBs and Bacteriophages

Computational models may play a crucial role in evaluating the potential synergistic effect of LBs and bacteriophages on meat safety and preservation. Predictive microbiology relies on the use of deterministic and stochastic models and is based on the idea that the responses of microbial populations to environmental elements can be replicated [54,55]. Thus, the application of predictive microbiology techniques allows for determination of the potential behavior of pathogens under diverse conditions within a certain degree of probability. The use of predictive biological models through databases with trained models offers a robust mechanism for enhancing quality support and ensuring food-safety measures in the food industry [56]. These models, grounded in evidence, signify progress in the effective evaluation and management of food safety [56].

**Figure 3:**
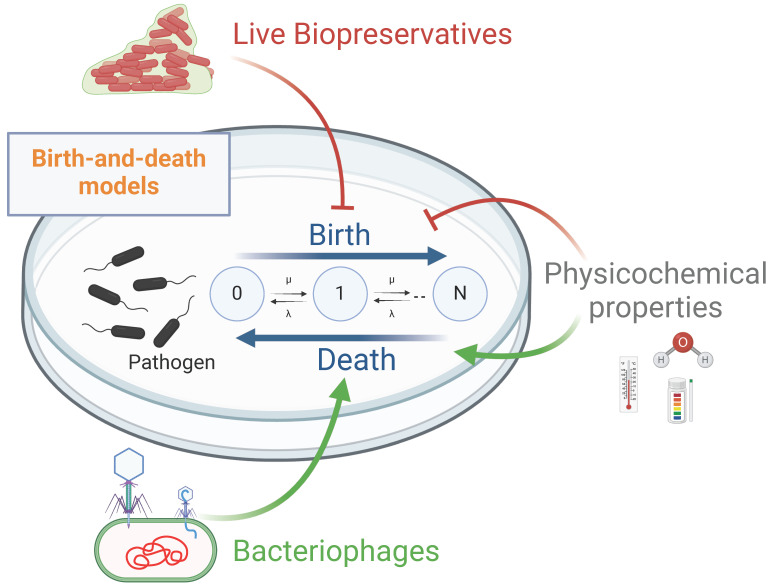
Hypothetical model of the action of live biopreservatives (LBs) and bacteriophages. Birth-and-death models for assessing the effect of LBs and bacteriophages on meat production. λ: birth rate and μ: death rate. The parameters (λ, μ) vary based on the microbes and experimental conditions (physicochemical properties, bacteriophages, and LBs). The synergistic interaction of LBs, bacteriophages, and the physicochemical properties of the food matrix, processing, and packaging on pathogen growth can be quantified using variations of the classic dynamic interaction equations (Equations (1)–(3)).

Counting models of pathogen growth can be written as general birth-and-death processes (Figure 3) [57]. Birth-and-death models focus on the internal dynamics of the size of a single population over time. These models offer versatility in their representations, allowing for diverse forms that encompass a wide spectrum of microevolutionary scenarios [58]. Nevertheless, birth-and-death models require a minimum of two parameters, λ (birth rate) and μ (death rate), to determine the effect of LBs and bacteriophages on pathogen growth. Thus, the population of a bacterial pathogen of size n increases at a rate of nλ and decreases at a rate of nμ, with the parameters (λ, μ) being different for each microbe and environmental condition.

Modelling interactions among a bacterial pathogen, an LB, a bacteriophage, and the physicochemical properties of meat production and storage can be achieved by combining aspects of competitive growth models and predator−prey dynamics based on the classic Lotka−Volterra equations [59] and the Monod and logistic growth models [60,61]. The concentration of the bacterial pathogen at time t, S(t), can be measured using Equation (1); the concentration of the LB at time t, B(t), can be measured using Equation (2); and the concentration of a bacteriophage targeting the pathogen at time t, P(t), can be measured using Equation (3). Furthermore, stochastic versions of these equations can account for real-world randomness, which is important for understanding population dynamics in small or variable populations.

Equation (1). Pathogen growth.

(1) $$\begin{array}{c} \frac{dS}{dt}=X_{S}-Y_{S}-Z_{S}\;\mathrm{when}\;X_{S}>Y_{S}+Z_{S}\\ X_{S}=\left(v\cdot \;i\right)\cdot S\cdot \left(1-\frac{S+B}{K}\right)\;Y_{S}=\gamma \cdot S\cdot B\;Z_{S}=\varphi \cdot S\cdot P\\ i=i_{t}\cdot i_{pH}\cdot i_{w}\cdot i_{m}\cdot \;i_{x}\;0\;\geq \;i\;\leq 1 \end{array}$$

where ν is the growth rate of the pathogen; K is the carrying capacity of the environment for bacteria; γ is the competition rate between B (LB) and S (pathogen); φ is the phage adsorption rate; and i is the interaction with environmental properties, including temperature (i_t_), water activity (i_w_), pH (i_pH_), material (i_m_), and other potential factors (i_x_).

Equation (2). Live biopreservative (LB) growth

(2) $$\begin{array}{c} \frac{dB}{dt}=X_{B}-Y_{B}\;\mathrm{when}\;X_{B}>Y_{B}\\ X_{B}=\left(\omega \cdot \;j\right)\cdot B\cdot (1-\frac{S+B}{K})\;Y_{B}=\alpha \cdot S\cdot B\\ j=j_{t}\cdot j_{pH}\cdot j_{w}\cdot j_{m}\cdot j_{x}\;0\;\geq \;j\;\leq 1 \end{array}$$

where ω is the growth rate of the LB; α is the competition coefficient between (S) pathogen and B (LB); and j is the interaction with environmental properties, including temperature (j_t_), water activity (j_w_), pH (j_pH_), material (j_m_), and other potential factors (j_x_).

Equation (3). Bacteriophage growth

(3) $$\begin{array}{c} \frac{dP}{dt}=X_{P}-Y_{P}\\ X_{P}=\beta \cdot \varphi \cdot S\cdot P\;\cdot (1-\frac{P}{K_{p}})\;Y_{P}=\delta \cdot P \end{array}$$

where β is the burst size (number of new phages produced per lysed host cell), δ is the natural decay rate of the phage, P is the bacteriophage concentration, and K_p_ is the carrying capacity of the environment for the bacteriophage.

### 8.3. Assessment of the Potential Impact of LBs on Antimicrobial Resistance

Antimicrobial resistance is a complex and emerging global health challenge characterized by the ability of microorganisms to withstand the effects of antimicrobial agents. The extensive utilization of antibiotics and other chemicals has the potential to induce multiresistance in microbes [62]. Horizontal gene transfer (HGT, i.e., the exchange of genetic material between different species) of antimicrobial resistance genes is a major source of antimicrobial resistance [63]. Thus, suitable LBs must not only exhibit the general characteristics outlined earlier in the text but must also be free from antimicrobial resistance genes. One may speculate that the use of LBs and bacteriophages could serve as an alternative to help mitigate the arms race. However, previous studies have shown that the use of antimicrobials may contribute to the formation of multiresistant bacterial strains [62,64,65,66]. Furthermore, the use of preservatives can accelerate plasmid-mediated HGTs of antimicrobial resistant genes (AMR) in bacteria [64]. Therefore, we hypothesize that interactions and competition within microbial communities, along with a potential increase in the frequency of HGTs, may accelerate the spread of AMR within a microbial community. In addition, considering that AMR mechanisms of bacterial resistance are interlinked [62], further investigation is needed into the potential impact of employing LBs and bacteriophages in meat production on the ongoing arms race of antimicrobial resistance [8] (Figure 2D).

### 8.4. Uses of LBs and Bacteriophages with Biodegradable Packaging for Meat

Active packaging or microencapsulation techniques are considered valuable alternatives for ensuring microbiological food safety and enhancing the quality of food products [34,67]. The application of antimicrobial and active compounds allows persistent migration to the food matrix during storage. In this regard, the application of these materials has various advantages, including controlling spoilage; restricting the growth of pathogenic microorganisms; reducing lipid, protein and pigment oxidation; and preventing off-odors [68]. Moreover, the incorporation of LAB and their metabolites in active packaging for meat and meat products is more efficient than their direct application [34]. However, to date, there are a limited number of studies that combine LBs [23] in sustainable/biodegradable food packaging for meat [48,49]. Some studies have shown the effectiveness of plantaricin BM-1 and pediocin BA-1/AcH for pork meat as coatings in new biocomposite films [1]. All the treatments reduced the population of *L. monocytogenes* by approximately 1.5–2 log units after 14 days of storage at 4 °C [1].

Even though the combination of different microencapsulation techniques, natural extracts, and LAB cocktails with novel techniques such as MAP, HPP, or active packaging increases the preservative effect [34], the synergistic potential of these combinations with bacteriophages in biodegradable materials has not yet been tested (Figure 2A). Moreover, biodegradable plastic packing is exposed to the action of environmental plastic-degrading microorganisms [69], leading to potential interactions with LBs and bacteriophages.

### 8.5. Socioeconomic Implications of LBs and Bacteriophages

The integration of LBs and bacteriophages in food-preservation processes has significant economic and environmental implications, reshaping the landscape of food safety and sustainability. Beyond technical considerations, addressing safety issues is crucial for ensuring the optimal and appropriate use of bacteriophages for maximum efficacy [15]; there is also the need for further legal revisions and weighting of ethical considerations. Bacteriophages are at the forefront of major innovations in biotechnology (e.g., as biopreservatives within the food industry [38]) and biomedicine (e.g., as phage therapy, a promising field of research with the aim of overcoming antibiotic resistance [70]). However, significant regulatory bodies in the EU and the United States have not overseen the utilization of bacteriophages in the fields of biomedicine and biotechnology [71]. Furthermore, consumers are not yet fully aware of the potential impact of these technologies. Although a limited recent survey in the United Kingdom showed that the lay public has a moderate level of acceptance for phage therapy [72], future, wider studies on public acceptance are still needed.

Economically, these natural preservatives offer a potential solution for industries where substantial financial losses are incurred due to product contamination [1]. By implementing biopreservation strategies, companies can potentially reduce waste, minimize recalls, and extend shelf life, leading to cost savings and improved profitability [31]. Research in this area aims not only at combating foodborne pathogens such as *L. monocytogenes* [33] but also at addressing critical environmental concerns by potentially curbing greenhouse gas emissions associated with food waste [31]. Moreover, the adoption of bioprotective starter cultures and other natural preservation methods can streamline production processes, reducing the reliance on synthetic additives and simplifying labeling, meeting consumer preferences for clean and natural ingredients (Figure 2B). This shift aligns with market demands for healthier, minimally processed foods, potentially enhancing market competitiveness for food producers [73]. For example, the implementation of higher concentrations of bioprotective starter cultures combined with low sugar concentrations has shown tangible benefits, such as improved textural qualities and reduced oxidation levels in products such as salamis, even when faced with challenges from various harmful bacteria, such as *L. innocua*, *S. enterica*, and *C. botulinum* [5]. Finally, the emergence of metagenomic techniques in food safety, particularly in identifying and characterizing environmental microorganisms in food-processing chains, represents a promising avenue for further investigation [1]. Such approaches not only enhance our understanding of food-preservation processes but also contribute to refining biopreservation strategies for better food quality and safety.

## 9. Conclusions

Biopreservation with LBs and bacteriophages is a promising sector of food processing and food research, with a specific spectrum of action against targeted bacterial species or even strains. Furthermore, biopreservation will be used in combination with physical factors such as heat treatment, chilling/cooling, or food drying, which results in a decrease in water activity. The emerging field of combining LBs and bacteriophages promises enhanced meat preservation and longevity. Adding LBs will yield meat products with an extended shelf life due to bacteriostatic mechanisms, whereas bacteriophages provide more direct (lytic) action against pathogens. However, achieving meat preservation with LBs and bacteriophages requires further empirical and theoretical understanding of these microbe-mediated strategies. A compiled list of outstanding questions regarding the use of LBs, bacteriophages, and biodegradable food packaging, to be addressed in future studies, is provided in Box 1. Moreover, the utilization of microbe-mediated approaches will create a need for additional revisions to the law and weighting of ethical considerations.

Box 1 **Outstanding questions**

- Is the combination of LBs (bacteriostatic) and bacteriophages (bacteriolytic) the most suitable strategy to improve quality and meat safety?
- What is the impact of combining LBs and bacteriophages on the physicochemical, technological, and sensory properties of both fresh and fermented meat?
- How do LBs and bacteriophages perform in real meat products, considering food complexities?
- How does the combination of LBs with biodegradable food packaging improve meat safety and sustainability?
- What potential interactions can occur between LBs (and bacteriophages) with environmental plastic-degrading microbes on biodegradable plastic packaging materials?
- Does the use of LBs (and bacteriophages) pose a potential risk for the spread of antimicrobial resistance?
- Can mixed strategies involving LBs and bacteriophages effectively address the issue of reducing nitrites and nitrates in meat preservation?

## Data Availability

Not applicable.
